# Wisdom teeth removal and anterior alignment stability after orthodontic treatment—a systematic review

**DOI:** 10.1007/s00784-026-06872-1

**Published:** 2026-04-24

**Authors:** Sophia Amberg, Annika Kroeger, Emma Houlston, Sinan Şen, Moritz Kebschull

**Affiliations:** 1 Department of Orthodontics, University Medical Center Schleswig-Holstein (UKSH), Kiel, Germany; 2 Department of Oral Surgery, Dentistry, School of Health Sciences, College of Medicine and Health, University of Birmingham, Birmingham, UK; 3 Birmingham Community Healthcare NHS Trust, Birmingham, UK; 4 Division of Periodontology and Oral Rehabilitation, Dentistry, School of Health Sciences, College of Medicine and Health, University of Birmingham, Birmingham, UK; 5 Division of Periodontics, Section of Oral, Diagnostic and Rehabilitation Sciences, Columbia College of Dental Medicine, Columbia University, New York, NY USA

**Keywords:** Wisdom teeth, Orthodontic treatment, Dental crowding, Anterior crowding, Post-treatment stability, Relapse, Systematic review

## Abstract

**Objective:**

Tertiary crowding is common after orthodontic treatment. The role of third molars in their development remains a subject of controversy. We aim to evaluate whether third molar removal affects long-term anterior alignment after orthodontic treatment.

**Material and methods:**

A systematic search was conducted in PubMed, Embase, Cochrane Library, and Google Scholar. Manual searches reference lists of included studies, selected orthodontic journals, and recent reviews. Included studies specifically addressed the following PICOT question: PICOT: In patients who do not wear retention devices after orthodontic treatment (P), does the extraction of wisdom teeth (I) compared to patients who retained their wisdom teeth (C) have an impact on the prevention of crowding (O) after at a minimum follow-up of one year after the end of the retention phase (T)?

Study selection, data extraction, and risk of bias assessment were performed independently by two reviewers.

**Results:**

Four studies met all inclusion criteria. All of them reported an increase in anterior crowding over time, regardless of third molar status. No statistically significant differences were found between groups. One study observed a smaller reduction in arch length in the extraction group. Overall, study quality was limited due to heterogeneity in treatment protocols, retention strategies, and measurement methods.

**Conclusion/clinical relevance:**

Based on the very low certainty of evidence, there is no consistent indication that the presence or agenesis/removal of third molars meaningfully influences post-treatment anterior crowding. Therefore, routine prophylactic extraction for this purpose is not supported by the currently available evidence.

Registration.

PROSPERO: CRD42024546851.

**Supplementary Information:**

The online version contains supplementary material available at 10.1007/s00784-026-06872-1.

## Introduction

Dental crowding is a prevalent issue in orthodontics, characterized by a mismatch between tooth dimensions and the available space for proper alignment [1]. It can be categorized as primary, secondary, and tertiary crowding. Tertiary crowding, also known as adolescent crowding, describes crowding in the area of the front teeth. This phenomenon occurs in both orthodontically treated and untreated patients and is the most common malocclusion [2, 3]. A population-based cross-sectional study revealed that the prevalence of anterior crowding is approximately 60% in the lower jaw and approximately 40% in the upper jaw [4]. Anterior crowding reduces smile harmony, facial aesthetics, and may have negative effects on the self-esteem and confidence of those affected [5]. Functionally, impaired cleaning properties, increased food impaction, and possible speech disturbances can arise. Resulting plaque accumulation and difficult oral hygiene subsequently increases susceptibility to dental decay and periodontal disease [6].

Tertiary crowding can occur as either a relapse or a developmental adjustment [7]. The precise causes of tertiary crowding remain unclear. It is assumed that several factors are responsible. Some authors attribute the reduction in dental arch width of the mandible as the primary cause, while others explain the development as a result of a combination of various factors [8]. These factors include the physiological mesial drift of the teeth, the pressure of soft tissue, the reduction of dental arch length, and further growth. The mandible grows longer than the maxilla, particularly in males, which is why many authors consider this to be a significant factor in the development of anterior crowding [9]. Furthermore, frequently observed rotation of the mandible and retrusion of the lower incisors can contribute to an enlarged overbite and the development of frontal crowding [10].

In addition to these various explanations, the question arises as to whether pressure from erupting wisdom teeth can also cause tertiary crowding. Multiple studies have yielded contradictory observations, leading to conflicting opinions among experts. Some authors conclude that pressure from erupting wisdom teeth indeed causes tertiary crowding [11–13], while others find no connection [14–16]. Explanations are centered on the idea that erupting or impacted third molars may exert mesial pressure on the posterior dentition, leading to forward movement of the arch and, ultimately, crowding of the lower incisors. However, contemporary evidence suggests that this mechanism is unlikely to explain anterior crowding, as mesial forces from third molars appear minimal and similar crowding changes are observed in individuals without third molars.

Compared with previous systematic reviews on this topic [17, 18], the present review applies more stringent and clearly defined inclusion criteria, including a minimum follow-up of one year after completion of the retention phase and a clearly specified main outcome measure (Little’s Irregularity Index). Furthermore, in contrast to earlier reviews, we also assessed maxillary alignment and additional outcomes such as intercanine width and arch length changes. By broadening the outcome scope and refining the eligibility criteria, the current review provides updated and more clinically relevant insights into the relationship between third molars and post-treatment alignment stability.

Therefore, this work aims to determine whether the removal of wisdom teeth/agenesis, compared to the retention of third molars, affects the prevention of tertiary crowding in patients after orthodontic treatment. We have conducted a systematic review, critically appraised included studies and their findings according to an a priori registered protocol.

## Materials and methods

### Study protocol and registration

The protocol, the review, and the manuscript were all designed and conducted according to the Cochrane Handbook for Systematic Reviews as a methodological guideline [19]. The study was registered a priori with the International Prospective Register of Systematic Reviews (PROSPERO reference CRD42024546851) [20]. The reporting of our findings adheres to the established PRISMA guidelines and checklist [21, 22].

### Research question

The research question was developed according to the PI/ECOT format (Population, Intervention/Exposure, Control, Outcome, and Time) [19].

In patients who do not wear retention devices after orthodontic treatment (P), does the extraction of wisdom teeth (I) compared to patients who retained their wisdom teeth (C) have an impact on the prevention of crowding (O) after at a minimum follow-up of one year after the end of the retention phase (T)?

### Inclusion criteria

- Comparative trials: randomized controlled trials and longitudinal observational studies (cohort studies)
- Written in English
  - The search was restricted to English language publications, as this was the only language in which all authors were fluent, ensuring accurate assessment of full-text methodological quality
- In vivo human studies
- Group size ≥ 20 participants/subjects per study group
  - The minimum group size was defined a priori to reduce the influence of small sample bias and unstable estimates in longitudinal analyses, and to ensure a minimum level of statistical robustness of the reported outcomes.
- Minimum follow-up of one year after the end of the retention phase
- Previously completed orthodontic treatment
- No wearing of removable retention appliances or fixed retainers after completion of the retention phase
- Wisdom teeth are present bilaterally in the upper or lower jaw and have been extracted bilaterally in either the upper, lower or both jaws.
- No additional format restrictions (e.g., publication type or file format) were applied beyond requiring peer-reviewed, full-text availability

### Exclusion criteria

Studies were excluded if they met any of the following conditions:

- Non-comparative study designs (e.g., cross-sectional studies, case series, case reports, reviews)
- Animal studies, in vitro studies, or studies not involving human participants
- Group sizes < 20 participants per study arm
- Follow-up shorter than one year after the end of the retention phase
- Ongoing use of fixed or removable retainers during the observation period
- Lack of previous orthodontic treatment
- Non-English publications
- Studies where full text was unavailable

### Search sources and strategy

The following electronic databases were searched for peer-reviewed publications (the most recent searches were conducted electronically on August 8, 2025):

- MEDLINE (Medical Literature Analysis and Retrieval System Online via PubMed)
- Embase
- Cochrane Library
- Google Scholar
  - Google Scholar was included to capture potentially relevant grey literature and non-indexed publications. All Google Scholar records retrieved through our search were screened using the same title/abstract review process applied to the major databases.

Manual search for eligible publications:

- January 2000 to August 2025; following journals: European Journal of Orthodontics (Volumes 22–47), The Angle Orthodontist (Volumes 70–95), and American Journal of Orthodontics and Dentofacial Orthopedics (Volumes 117–168)
- The reference lists of all eligible full texts were searched for additional potentially relevant articles
- Latest reviews
- Experts in the topic: As part of the manual search, two subject experts within the author team (A.K. and S.S.), both with extensive clinical and academic experience in orthodontics and oral surgery, were asked whether they were aware of any additional published studies meeting the predefined eligibility criteria. Both confirmed that they knew of no further relevant publications.

For search terms, please refer to Supplementary Table 1.

### Study selection

All retrieved records were imported into a reference management software Endnote (Clarivate, Philadelphia, PA). Duplicate records were initially identified and removed using the software’s automated duplicate detection function. A subsequent manual verification step was performed to ensure complete and accurate removal of remaining duplicates prior to title and abstract screening.

Two independent reviewers screened titles and abstracts (SA, EH). Studies were included if both reviewers agreed that they potentially met all inclusion and exclusion criteria. An initial calibration was performed, and inter-rater reliability (percentage of agreement) was calculated. If abstracts were inconclusive, full texts were obtained for further review.

Any disagreements were referred to a third reviewer (AK) for discussion until agreement was reached. In the next step, the two reviewers (SA, EH) independently conducted a full-text review. If studies met all inclusion criteria and no exclusion criteria, they were included. Any disagreements were discussed with a third reviewer (AK) to reach a consensus and finalize the decision.

Publications excluded at this stage have been documented, including the justification of exclusion (Supplementary Table 2).

### Data extraction

Two reviewers (SA, EH) independently performed duplicate data extraction using a predetermined spreadsheet. In cases of missing or incomplete data, clarification was sought from the study authors. In addition to the predefined outcomes, the following data items were extracted: study design, study setting, sample size, study groups and their definition, patient demographics (age and sex where reported), retention duration, study visits, follow-up duration, measurement methods, and reported statistical results. When dealing with studies involving multiple arms or multiple papers reporting on the same study, only one set of relevant data was extracted. Any disagreements in decisions were addressed after consulting with the third reviewer (AK).

### Outcomes

The main outcome chosen is the degree of frontal crowding in the form of contact point discrepancies (mm), measured using the Little Irregularity Index. This index quantifies the sum of deviations between the anatomic contact points of the six mandibular anterior teeth by adding the horizontal distances between adjacent contact points [23].

The additional outcomes were:

- Change in intercanine distance (mm)
- Change in arch length (mm)
- Change in overbite and overjet (mm)
- Change in position and inclination of mandibular incisors (mm)
- Change in position of the first molars (mm)
- Patient-related outcomes: pain and quality of life

### Quality assessment

Two reviewers (SA, EH) assessed risk of bias using specific tools: the RoB 2.0 tool for randomized controlled trials [24] and the Newcastle–Ottawa Scale (NOS) for observational studies [25]. Any discrepancies between the reviewers were resolved by seeking the opinion of a third reviewer (AK).

### Narrative synthesis of findings

As meta-analyses were not feasible due to substantial clinical and methodological heterogeneity, a structured narrative synthesis was conducted in accordance with guidance from the Cochrane Handbook [19]. Studies were grouped by predefined outcomes (e.g., anterior alignment, intercanine width, arch length), and findings were summarized study-by-study. Patterns, consistencies, and discrepancies across studies were compared qualitatively. The risk of bias of all included studies was assessed and described in detail.

### Assessment of the overall certainty of outcomes—GRADE

An outcome-level certainty assessment was conducted using the GRADE framework as described in the Cochrane Handbook [19].

## Results

### Study selection

**Fig. 1:**
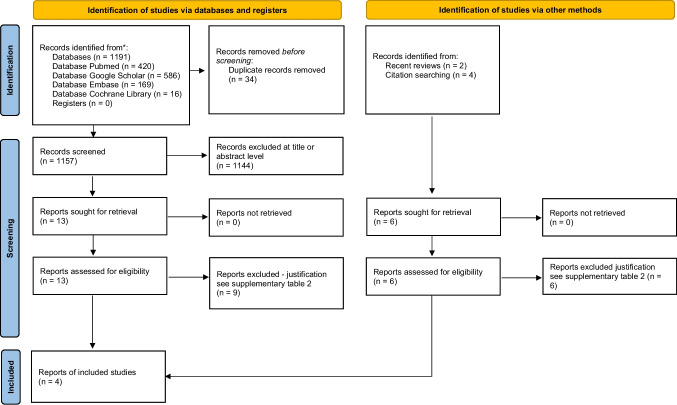
Prisma flow chart

The electronic search identified a total of 1191 studies. After removing duplicates, 1157 records remained. After title and abstract screening, 13 full texts were assessed for eligibility. After searching the reference lists of all eligible full texts for additional potentially relevant articles and reviewing the most recent systematic reviews, a final selection of four individual studies was made (see Fig. 1: PRISMA flowchart). A list of articles excluded at full-text stage screening, together with the reasons for their exclusion, can be found in Supplementary Table 2.

### Inter-rater agreement

The inter-rater raw agreement for title and abstract screening was 99.54%. The kappa correlation coefficient for the risk of bias analyses was k = 1, indicating a very good level of agreement. In addition, the inter-rater agreement was 100%.

### General details and study design of the included studies

#### General characteristics

A total of four studies met all inclusion criteria and none of the exclusion criteria, and were therefore selected. These studies initially involved a total of 468 patients [26–29]. One of the four studies was a randomized controlled trial, while the remaining three were retrospective longitudinal cohort studies. All articles were published between 1990 and 2020. At the outset of the study, the maximum number of patients enrolled was 164, while the minimum number was 97. An overview of the general study characteristics can be found in Table 1.

**Table 1:** Detailed studies’ description

Authors	Setting	Study groups	Sample size—total participants at study start	Sample size—total participants at end of study	Age mean (Range) or mean ± SD in years	Gender distribution (% females)	Study group definition	Outcomes	Measurement method	Studytype	Time	Mean postretention time
Ades, A.G 1990	clinic and Practice	control group	46	38	T3: 28.5 years (18.5—39.3 years)	NI	1. M3 bilaterally erupted 2. M3 bilaterally impacted	1. Irregularity Index (Little), 2. Mandibular Intercanine Distance, 3. Mandibular Arch Length, 4. Overbite, 5. Overjet, - FRS Analysis	Plaster models measured with Helios calipers (measurement accuracy 0.1 mm), measurement conducted by one person	Retrospective cohort study	T1: before orthodontic treatment, T2: after orthodontic treatment, T3: after retention	13 years (10—28 years)
		test group	51	46			1. Exposure: Agenesis of M3 bilaterally 2. Intervention: Bilateral extraction of M3 at least 10 years before time point T3					
Cotrin 2020	Clinic	control group	72	72	Group 1: Patients with Erupted or Impacted Mandibular Third Molars T1: 13.12 ± 1.02 years T2: 15.26 ± 1.12 years T3: 20.67 ± 1.30 years	54.2	1. M3 bilaterally erupted 2. M3 bilaterally impacted	1. Irregularity Index (Little), 2. Overbite	Measurements of the models using a digital precision caliper with an accuracy of 0.01 mm (Mitutoyo America, Aurora, Illinois)	Retrospective cohort study	T1: before orthodontic treatment, T2: after orthodontic treatment, T3: at least 3 years after retention	Group 1: Patients with Erupted or Impacted Mandibular Third Molars 5.41 ± 1.04 years
		test group	36	36	Group 2: Patients with Agenesis or Extraction of Mandibular Third Molars T1: 13.37 ± 1.27 years T2: 15.75 ± 1.41 years T3: 20.89 ± 1.84 years	50	1. Exposure: Agenesis of M3 bilaterally 2. Intervention: Bilateral extraction of M3					Group 2: Patients with Agenesis or Extraction of Mandibular Third Molars 5.14 ± 1.10 years
Harradine 1998	Clinic	control group	164 (Test group: NI, Control group: NI)	33	T1: 14.83 ± 1.35 years	54.88% (Test group: NI, Control group: NI)	M3 bilateral not extracted	1. Irregularity Index (Little), 2. Intercanine Distance, 3. Arch Length in the maxilla and mandible	Models were digitized, analysis conducted using the statistical software MinitabTM and GLIM	Randomized controlled trial	T1: after the end of retention, T2: at least five years after entering the study	5.5 ± 1.05 years
		test group		44			Bilateral extraction of M3					
Van der Schoot E.A. 1997	Clinic	control group	88 (each jaw was judged separately)	NI	T1: 12.8 ± 3.10 years T2: 15 ± 2.90 years T3: 22.3 ± 4.20 years	60,61% (Test group: NI, Control group: NI)	1. M3 bilaterally erupted 2. M3 bilaterally emerged	1. Irregularity Index (Little), 2. Arch Length Discrepancy of the upper and lower anterior teeth, 3. Arch Length Discrepancy of the premolar area on the left and right	Measurements on plaster models	Retrospective cohort study	T1: before orthodontic treatment, T2: after orthodontic treatment, T3: at least 3 years after retention	NI
		test group	95 (each jaw was judged separately)				1. Exposure: Agenesis of one or both M3s 2. Intervention: Bilateral extraction of M3					

*y *years, *SD *standard deviation, *NI *no information

In the cohort studies, measurements were conducted at three different time points: T1 was prior to orthodontic treatment, T2 occurred immediately after orthodontic treatment, and T3 took place following the completion of the retention period.

In the randomized controlled trial, measurements were taken at two distinct time points: T1 was after orthodontic treatment, and T2 occurred at least five years after enrolment in the study. While the randomized controlled study considered only whether wisdom teeth are present, the other studies occasionally differentiate between impacted, unerupted, and erupted wisdom teeth.

Average follow-up varies between studies, with a maximum of 13 years and a minimum of 5.14 years. Detailed descriptions of the characteristics are presented in Table 1.

Across the included studies, patients generally wore retainers during the immediate post-treatment retention phase. These appliances were removed prior to the start of the follow-up period from which outcome data were extracted. The duration and type of retention were not consistently reported. Ades et al. (1990) stated that all participants were free of retention for at least ten years; Harradine et al. (1998) enrolled patients only after they had completed their retention phase; Cotrin et al. (2020) specified a mandibular canine-to-canine bonded retainer worn for one year and removed before the post-retention assessment; and Van der Schoot et al. (1997) evaluated patients at least three years after the end of retention.

#### Risk of bias

For the cohort studies, Ades et al. (1990) received a score of 6/9, indicating a moderate risk of bias. The study had good selection criteria and a valid assessment of exposure. It performed poorly in terms of comparability, and it was unclear whether the endpoints were assessed validly and independently, such as through independent or blinded assessment by multiple investigators. Cotrin et al. (2020) scored 8/9, suggesting a low risk of bias. This study was strong in terms of comparability. However, it was unclear whether the endpoints were assessed in a blinded manner. In contrast, Van der Schoot et al. (1997) scored 5/9, reflecting a moderate risk of bias, with particular weaknesses in comparability and handling missing data.

The randomized controlled trial (RCT) by Harradine et al. (1998) was assessed using the RoB 2.0 tool and found to have a high risk of bias. This was primarily due to missing outcome data, with only 47% of participants completing the study (Tables 2 and 3).

**Table 2:** Quality assessment of cohort studies

Studies/Protocols	Selection				Comparability	Outcome			Summary judgement
	1. Is the exposed cohort representative of the intervention/exposure being studied?	2. Is the non-exposed cohort representative, and was it adequately selected?	3. Was there a valid assessment of exposure?	4. Is it likely that the measured endpoint was not present at the start of the study?	1. Is there comparability between the exposed and non-exposed cohorts?	1. Was there a valid assessment of endpoints?	2. Could the endpoint occur during the observation period?	3. Were missing data adequately considered?	
Authors, year	(0), (1)	(0), (1)	(0), (1)	(0), (1)	(0), (1), (2)	(0), (1)	(0), (1)	(0), (1)	
Ades et al., 1990	1	1	1	1	0	0	1	1	6
Cotrin et al., 2020	1	1	1	1	2	0	1	1	8
Van der Schoot et al., 1997	1	1	1	1	0	0	1	0	5

*y* years, *SD* standard deviation, *NI* no information

**Table 3:** Quality assessment for the randomized trial

Domain	Study: (Harradine et al., 1998)	Risk-of-bias judgement
D1—Risk of bias arising from the randomization process	1.1 Was the allocation sequence random? - Yes.—“A list of randomly generated numbers was used to allocate extraction or retention of the third molars for that patient.” 1.2 Was the allocation sequence concealed until participants were enrolled and assigned to interventions? - Yes 1.3 Did baseline differences between intervention groups suggest a problem with the randomization process? - No	**Low**
D2—Risk of bias due to deviations from the intended interventions	2.1 Were participants aware of their assigned intervention during the trial? - Yes 2.2 Were carers and people delivering the interventions aware of participants' assigned intervention during the trial? - Yes, 2.3 Were there deviations from the intended intervention that arose because of the trial context? - No information The investigators and surgeons were aware of the allocation of patients. The study participants were aware of the planned intervention. No appropriate analysis was used to calculate the effects of intervention allocation	**Some concerns**
D3—Missing outcome data	3.1 Were data for this outcome available for all, or nearly all, participants randomized? - No. Of 164 patients, 77 patients completed the study after five years. The exact reasons for this are not known. This means that 47 per cent of the original study participants actually completed the study 3.2 Is there evidence that the result was not biased by missing outcome data? - No 3.3 Could missingness in the outcome depend on its true value? - Probably yes 3.4 Is it likely that missingness in the outcome depended on its true value? - Probably yes. However, the authors categorise the risk of this bias as low: “Generalized linear modelling was carried out resulting in the initial casts of 44 of the non-responders being digitized to ascertain if any responder bias was present with respect to the outcome measurements. All values fell within the 96 per cent confidence intervals and it was concluded that no systematic differences occurred between those patients who entered the trial and completed and those who entered and did not complete.”	**High**
D4—Risk of bias in measurement of the outcome	4.1 Was the method of measuring the outcome inappropriate? - No 4.2 Could measurement or ascertainment of the outcome have differed between intervention groups? - No 4.3 Were outcome assessors aware of the intervention received by study participants? - No. “A jig was designed in order to practice the digitizing technique, calibrate the digitizer, and to assess both precision and reproducibility. The third molar status was unknown to the digitizer in order to eliminate sub-conscious bias. Approximately 10 per cent of records were redigitized after an interval of at least 3 months in order to calculate the error of the method. The mesial and distal anatomical contact points of all teeth from the first molars mesially in both jaws were digitized and the three-dimensional co-ordinates stored.“	**Low**
D5—Risk of bias in selection of the reported result	5.1 Were the data that produced this result analysed in accordance with a pre-specified analysis plan that was finalized before unblinded outcome data were available for analysis? - Probably yes. “The programme was used to calculate Little’s two-dimensional Index of Irregularity (Little, 1975) intercanine width and arch length in both the upper and lower arches.” 5.2 Is the numerical result being assessed likely to have been selected, on the basis of the results, from multiple eligible outcome measurements (e.g. scales, definitions, time points) within the outcome domain? - No 5.3 Is the numerical result being assessed likely to have been selected, on the basis of the results, from multiple eligible analyses of the data? - No	**Low**
In total	The study is considered to be at high risk of bias in at least one area	**High**

*y* years, *SD* standard deviation, *NI* no information

Due to the high heterogeneity in study designs, outcomes measured, and the generally poor risk-of-bias scores, a meta-analysis could not be performed.

#### Main outcome

Regarding the main outcome, all studies showed similar results. There was no statistically significant difference in the amount of anterior crowding, as measured by the Little irregularity index, between the test and control groups during the follow-up period in any of the included studies. In the test group, the patients had their third molars (M3) extracted, while the control groups consisted of patients whose M3 were either impacted, erupted, or simply emerged. It is essential to note that in some studies, the control group was further divided into subcategories (impacted, erupted, and emerged wisdom teeth) to assess whether the different states of M3 had different effects on the results.

Specifically, Ades et al. (1990) reported an increase in crowding of 3.25 mm (SD = 5.34) in the M3 extracted group, compared to 2.27 mm (SD = 1.81) in the M3 impacted group and 3.19 mm (SD = 2.2) in the M3 erupted group, but no statistically significant differences were reported between test and control groups. Similarly, Cotrin et al. (2020) found an increase in crowding of 1.96 mm (SD = 2.47) in the M3 extracted group, compared to 1.38 mm (SD = 2.05) in the M3 impacted group and 1.73 mm (SD = 1.22) in the M3 erupted group, again with no statistically significant differences. Harradine et al. (1998) observed an increase in crowding of 0.8 mm (SD = 1.23) in the M3 extracted group and 1.1 mm (SD = 2.72) in the M3 non-extracted group, with no significant differences noted. Lastly, Van der Schoot et al. (1997) reported an increase in crowding in all groups (M3 extracted, M3 erupted, and M3 emerged), but no statistically significant differences were observed between the groups. The detailed main outcome of each study is presented in Table 4.

**Table 4:** Main results of included studies

Authors	Test groups	Crowding (mm)—Test groups	Control groups	Crowding (mm)—Control groups	Results
Ades et al., 1990	M3Extracted	3.25, SD = 5.34	M3Impacted	2.27, SD = 1.81	No statistically significant result
			M3Erupted	3.19, SD = 2.2	
Cotrin et al., 2020	M3Extracted	1.96, SD = 2.47	M3Impacted	1.38, SD = 2.05	No statistically significant result
			M3Erupted	1.73, SD = 1.22	
Harradine et al., 1998	M3Extracted	0.8, SD = 1.23	M3Non-Extracted	1.1, SD = 2.72	No statistically significant result
Van der Schoot et al., 1997	M3Extracted	Increase in crowding	M3Erupted	Increase in crowding	No statistically significant result
			M3Emerged	Increase in crowding	

*y* years, *SD* standard deviation, *NI* no information

#### Secondary outcomes of included studies

Similar to the main outcome, there were few statistically significant differences between the test and control groups during the follow-up period for the secondary outcomes.

##### Arch length

Three trials analyzed changes in arch length [26, 27, 29]. Harradine et al. (1998) found a statistically significant difference in arch length between the M3 extracted group (−1.1 mm, SD = 1.13) and the M3 non-extracted group (−2.13 mm, SD = 0.97), with a significantly smaller reduction in the M3 extracted group. Both other studies reporting on arch length changes did not find any statistically significant differences between groups.

##### Intercanine distance

Changes in intercanine distance were investigated in some studies [26, 27], but no significant differences were identified between the test and control groups.

##### Overbite and overjet

Ades et al. (1990) examined changes in overbite and overjet, but no significant differences were observed between the test and control groups.

##### Incisor and molar positions

Ades et al. (1990) also assessed the positions of the lower incisors and first molars, as well as the inclination of the incisors, using lateral cephalometric radiographs. Again, no statistically significant differences were found between the groups.

The detailed secondary outcomes of each study are presented in Supplemental Table 3.

##### Patient-reported outcomes

No included studies reported any patient-related outcomes (e.g., pain, quality of life, satisfaction), and therefore, they could not be meaningfully synthesized.

### GRADE outcome certainty assessment

Based on the GRADE assessment, all outcomes were rated as very low certainty due to study heterogeneity, imprecision, and risk of bias. An overview of the assessment outcome can be found in Table 5.

**Table 5:**
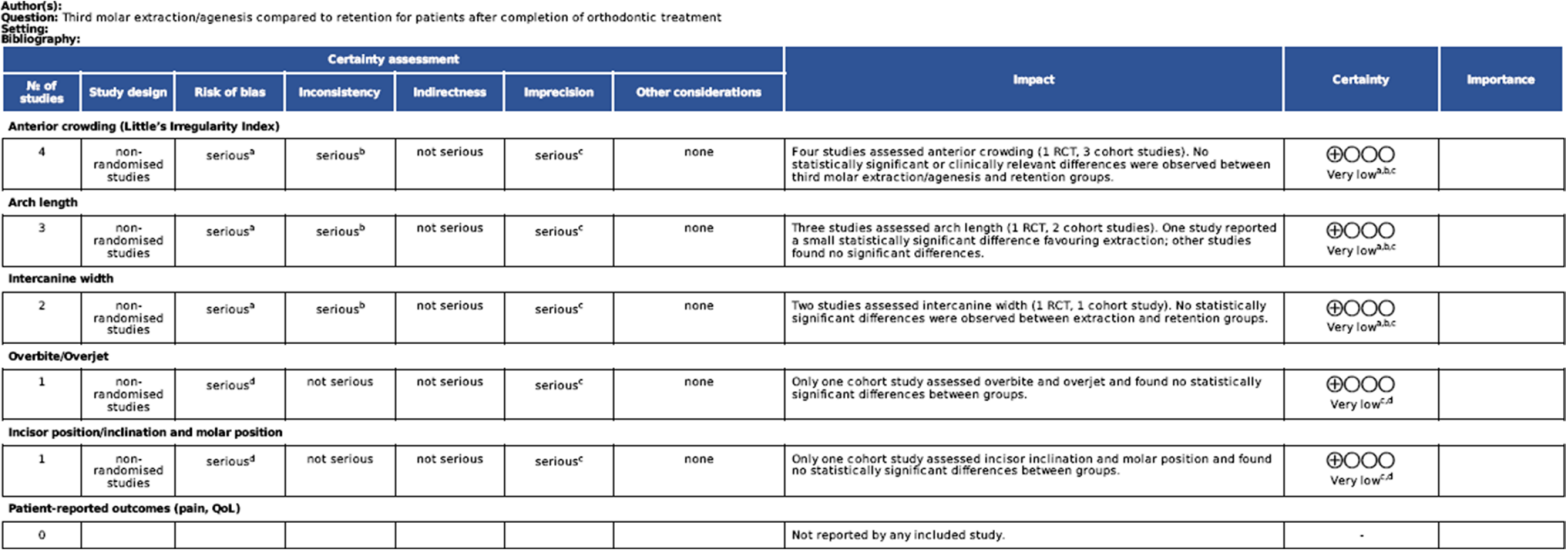
GRADE outcome certainty assessment

*CI* confidence interval

Explanations

a. Downgraded one level due to moderate limitations in cohort studies and high attrition with risk ofbias in the included RCT.

b. Downgraded one level due to substantial clinical and methodological heterogeneity across studies, including differences in trestment protocols, retention strategies, and folloe-up duration.

c. Downgraded one level due to small sizes, limiting confidence in the abscene of a true effect.

d. Downgraded due to moderate methodological limitations of the included study.

## Discussion

### Interpretation of findings

The present systematic review aims to investigate the impact of wisdom teeth removal on long-term stability following orthodontic treatment, with a particular focus on the development of crowding in the anterior teeth, which may be influenced by the presence of wisdom teeth.

Across all included studies, both patients with and without wisdom teeth experienced an increase in anterior crowding after orthodontic treatment. Harradine et al. [27] reported a slightly lower mean increase in crowding for patients without wisdom teeth (0.8 mm) compared to those with wisdom teeth (1.1 mm); however, this difference was minor and not statistically significant. In contrast, two studies found a slightly higher increase in crowding in patients who had undergone wisdom tooth removal, yet again, these differences were small and statistically insignificant [26, 28]. The study by Van der Schoot et al. [29] provided no precise numerical data but confirmed a general increase in crowding among all patient groups during the follow-up period.

Similar to the primary outcome, the secondary endpoints showed few statistically significant differences between the groups. Only Harradine et al. [27] observed a meaningful difference, reporting a significantly smaller decrease in arch length in the extraction group (−1.1 mm, *SD* = 1.13) compared to the non-extraction group (−2.13 mm, *SD* = 0.97). For other parameters such as intercanine width, overbite, overjet, and cephalometric measurements, no significant differences were detected.

### Comparison to existing literature

The present findings are consistent with those of two recent reviews examining the influence of wisdom teeth on anterior crowding [17, 18]. While Pithon et al. [18] focused primarily on crowding of the mandibular incisors after orthodontic treatment, the present review also considers the maxillary anterior teeth. Three of the studies analysed here were likewise included in the review by Pithon et al., whereas three others were excluded from the current analysis for various reasons. One of these was the study by Okazaki et al. [30], which investigated interproximal forces rather than crowding and was therefore not eligible. Interestingly, this was the only study to report a significant association between the presence of wisdom teeth and increased force levels—particularly in patients with severe pretreatment crowding and beyond six months of retention. However, most of the studies included in this review did not clearly specify the initial severity of crowding, and some of the reported outcomes refer to periods after the retention phase had already ended.

In the recent review by Lyros et al. [17], a total of ten studies were analysed. Four of the studies included in the present review were also part of that analysis. The remaining six were excluded from this review for various reasons. This discrepancy is due to differences in the inclusion and exclusion criteria between the two reviews. Some of the studies analysed by Lyros et al. were already excluded at the title and abstract screening level in the present review, while others were excluded after full-text screening, as shown in Supplementary Table 2. Two of the excluded studies reported statistically significant associations between the presence of wisdom teeth and the development of anterior crowding [30, 31]. In the study by Kahl-Nieke et al. [31], a slightly higher irregularity index was observed in patients with wisdom teeth. However, the difference was minimal and likely of limited clinical relevance. The majority of studies analysed by Lyros et al. [17] found no statistically significant differences between the test and control groups.

There are, however, individual studies that suggest a possible influence of wisdom teeth on anterior crowding. One such study was conducted by Lindqvist and Thilander [32], who found that unilateral extraction of third molars led to improved space conditions on the extraction side in 70% of cases. Based on these findings, the authors recommended extraction in cases of severe crowding. However, the study design differs substantially from those included in the present review, making direct comparison difficult. The test and control groups were defined by sides of the same jaw, and outcomes were measured in terms of arch length rather than using Little’s irregularity index.

Other studies—such as those by Abdulla et al. [33], Richardson et al. [34], and Vego et al. [11]—have also reported an association between third molars and anterior crowding. However, the patients in these investigations had not received orthodontic treatment. As a result, these studies are likewise not directly comparable to those included in the present review.

In addition to the available evidence, some clinicians question whether third molars are the main cause of late anterior crowding. Tosun [35], for instance, suggests that these teeth are structurally too weak to exert enough force to displace the anterior dentition. In cases of limited space, they often remain impacted or erupt towards areas of least resistance, rather than pushing through the dental arch. He argues that factors such as mesial migration, age-related soft tissue changes, and functional imbalances are more likely to explain late crowding—even in the absence of wisdom teeth. This supports the view that post-treatment crowding is multifactorial in nature.

### Limitations of the current review

Several limitations of the included studies need to be taken into account when interpreting the results. A key limitation of this review lies in the heterogeneity of the patient populations. The most detailed inclusion criteria were reported by Ades et al. [26] and Cotrin et al. [28]. Ades et al. [26] included only patients with a post-treatment irregularity index below 3.5, and excluded those with prior circumferential supracrestal fiberotomy or carious teeth after active treatment—criteria not specified in the remaining studies. Cotrin et al. [28] excluded patients with anomalies in tooth morphology or those who had undergone interproximal enamel reduction during orthodontic therapy.

Information on the initial severity of crowding before treatment would have been helpful to assess the risk of relapse more precisely. However, this was not reported in most studies; only Cotrin et al. [28] provided this data. Similarly, the post-treatment alignment of the anterior teeth was rarely described, even though it may influence the stability of the treatment outcome.

Another important limitation is the considerable heterogeneity in the initial treatment approaches. Although all patients in the included studies received orthodontic treatment, the modalities varied significantly. In some cases, premolars were extracted, while others were treated with removable appliances or full fixed multibracket systems. Such differences, particularly space-altering interventions like extractions, can influence post-treatment stability and affect the risk of relapse. Furthermore, it remains unclear whether biological limits—such as the intercanine width—were respected during treatment. The absence of this information further limits the comparability of results across studies.

In many of the included studies, the retention phase was poorly documented. It often remained unclear whether patients wore fixed or removable retainers and for how long. The timing of the final follow-up point (T3) also varied considerably: some studies defined it as the end of the retention period, while others set it after retention had already ended. These inconsistencies reduce the comparability of the studies and complicate the interpretation of long-term outcomes.

The timing of third molar removal is another factor that may have influenced the outcomes. Of all included cohort studies, only Ades et al. [26] reported this information in detail, stating that extractions had taken place at least ten years prior to the final follow-up (T3). In the remaining studies, the exact timing of extraction was not specified. Furthermore, the cohort studies failed to provide detailed information on the indication for extraction, the preoperative status of the third molars, any potential molar pathologies, or the conditions of the retromolar space. This lack of information may have a considerable impact on the interpretation of the results.

There were notable differences in how measurements were conducted across the included studies. Two studies used plaster models and manual calipers with varying levels of precision [26, 28], while only one study relied on digital models and software [27]. In the study by van der Schoot et al. [29], the models were assessed on plaster casts, but no further methodological details were provided. In most studies, it was not specified whether measurements were performed in a blinded manner, which could have introduced observer bias. None of the included studies reported statistically significant differences between groups. The excluded study by Kahl-Nieke et al. [31] found a statistically significant difference of 1.2 mm in favor of patients without third molars. However, the authors themselves questioned the clinical relevance of this result, pointing out that the observed difference was small and fell within the reported range of measurement error (0.3–1.0 mm). Against this background, minor variations in crowding should be interpreted with caution, particularly when considered as a justification for third molar removal in the absence of other clinical indications.

The only randomized controlled trial [27] included in this review was rated as having a high risk of bias, mainly due to significant attrition. The authors explained the dropout by stating that participants had moved away and could no longer be reached through their general or dental practitioners.

Patients who had previously undergone premolar extractions were only eligible if all extraction spaces were fully closed at baseline. However, this criterion was not consistently applied: in a substantial number of cases, residual spaces were still visible on the initial models. Although this issue was identified retrospectively and the affected patients were subsequently excluded, no justification was provided for the initial deviation from the protocol. These additional exclusions reduced the final sample to only 33% of the originally recalled patients, which further increases the risk of attrition bias.

Despite these methodological concerns, the study was cited in the current German clinical guideline [36], particularly in reference to the statistically significant difference in arch length between the extraction and non-extraction group. However, given the study’s limited quality, the generalizability of this result is questionable.

All in all, this work highlights the lack of randomized controlled trials or high-quality cohort studies that provide convincing clinical and statistically significant results to justify such an invasive procedure as third molar removal. Stratification based on the type of third molar impaction may be considered. Additionally, questions remain regarding how the randomization process can be ethically and practically implemented in future studies, particularly in patients without a convincing clinical indication for wisdom tooth removal.

### Clinical context

The indication for the prophylactic removal of wisdom teeth remains a topic of debate. While some studies have suggested a potential link between wisdom teeth and the development of anterior crowding, the findings of this systematic review show no statistically significant relationship between the presence of wisdom teeth and the development of anterior crowding after successful completion of orthodontic treatment. Given this, the connection between wisdom teeth and crowding appears weak and inconsistent, making prophylactic removal based solely on this rationale questionable. Whilst our work does not support the surgical removal of third molars solely for stabilisation of anterior alignment, several other orthodontic considerations (such as posterior occlusal changes or positional changes of second molars in the presence of a third molar) may influence decision-making regarding third molar extraction.

Further information on the type of impaction is missing. Important stratification and risk factors may be missed. Recent studies show an increased risk of decay on neighboring molars on mesio-angular impacted teeth, compared to other types of impaction [37]. The incidence of distal molar decay adjacent to impacted wisdom teeth increases with increasing age of the patient [38]. Similar patterns may be present in these research questions, but have not been investigated.

Finally, patients should be given optimal advice. On the one hand, repeat treatment due to crowding should be avoided, and on the other hand, it must be remembered that the removal of wisdom teeth is an invasive procedure. Several complications can arise from the surgical removal of wisdom teeth. These include postoperative infections, nerve damage, particularly to the lingual and inferior alveolar nerve, damage to adjacent teeth, sinus perforations, bleeding complications, anesthesia-related injuries, jaw fractures, and postoperative pain and swelling [39–41]. Given these short, mid-, and potentially long-term risks, the decision to extract wisdom teeth should be made with caution and with sound justification and indication. The therapeutic benefits must clearly outweigh the associated risks, and such decisions should be based on individual patient factors rather than generalized prophylactic measures.

## Conclusions

The current review demonstrates that, based on very low certainty of evidence,there is no consistent evidence to suggest that wisdom tooth removal positively influences long-term stability of anterior alignment following orthodontic treatment. The results of the included studies show no statistically significant differences between the groups. The considerable heterogeneity in the data and the numerous biases present in the studies make it challenging to draw meaningful comparisons and provide a definitive answer to the research question posed by this systematic review. To consider wisdom tooth removal from an orthodontic perspective, especially under consideration of associated risks, further clear and homogeneous data are required based on sufficient sample sizes.

## Supplementary Information

Below is the link to the electronic supplementary material.

Supplementary file1 (DOCX 22 KB)

Supplementary file2 (XLSX 11 KB)

Supplementary file3 (XLSX 12 KB)

## Data Availability

There are no new data associated with this article.
