# Supplementary material for: Wisdom teeth removal and anterior alignment stability after orthodontic treatment—a systematic review
Source: Clin Oral Investig. 2026 Apr 24;30(5):199. doi: 10.1007/s00784-026-06872-1 (PMC13109118; doi:10.1007/s00784-026-06872-1)
Supplement: Supplementary file 1 — Supplementary file1 (DOCX 22 KB) [file 784_2026_6872_MOESM1_ESM.docx]

Supplementary Table 1: Search Terms

**1.Embase:**

| #1 | third molar or wisdom |
| --- | --- |
| #2 | (ortho* or brace*) |
| #3 | (crowd* or align*) |
| #4 | #1 AND #2 AND #3 |

**2.Cochrane:**

| #1 | MeSH descriptor: [Molar,Third] explode all trees |
| --- | --- |
| #2 | MeSH descriptor: [Orthodontics] explode all trees |
| #3 | #1 AND #2 |

**3. Pubmed:**

| #1 | Molar,Third[Mesh] AND Orthodontics[Mesh] |
| --- | --- |

**4. Google Scholar:**

| #1 | (("third molar extraction" OR "third molar removal" OR "wisdom tooth removal" OR "wisdom tooth extraction" OR "wisdom teeth removal" OR "wisdom teeth extraction") AND (orthodontics OR orthodontic) AND crowding AND stability) |
| --- | --- |
